# Navigating Immunotherapy Resistance: The Role of Cross-Line Strategies in Cancer Treatment

**DOI:** 10.3390/jcm15072751

**Published:** 2026-04-05

**Authors:** Yan Kang, Hongchao Zhen, Jing Wang, Haishan Lin, Bangwei Cao

**Affiliations:** 1 Department of Oncology, Beijing Liangxiang Hospital, Capital Medical University, No. 45 Gong Chen North Street, Funhill District, Beijing 102400, China; kamkang_china@hotmail.com; 2 Department of Oncology, Beijing Friendship Hospital, Capital Medical University, No. 95 Yong An Road, Xicheng District, Beijing 100050, China; zhchao116@163.com (H.Z.); 18911188803@163.com (J.W.); linhaishan2199@sina.com (H.L.)

**Keywords:** cross-line immunotherapy, D-dimer, hypertriglyceridemia, hyponatremia, immune-related events

## Abstract

**Background/Objectives:** Cancer is the second leading cause of death globally, and resistance to immunotherapy requires new strategies. One promising approach is cross-line immunotherapy, defined as retreatment with the same or different immune checkpoint inhibitors after progression on prior immunotherapy. Understanding the efficacy and safety of this innovative treatment modality is critical for advancing cancer care. **Methods:** In this study, we evaluated outcomes of cross-line immunotherapy in a cohort of 105 patients with various malignant tumors at Beijing Friendship Hospital. The primary endpoints of the study included progression-free survival (PFS2) and overall survival (OS), measured from the initiation of cross-line immunotherapy. All patients received treatment regimens determined by their physicians. The study aimed to evaluate the efficacy of cross-line immunotherapy, with or without other therapies. **Results:** The study reported a median PFS2 of 6.9 months (95% CI: 4.7–8.1) and a median OS of 12.9 months (95% CI: 11.7–NA). The objective response rate (ORR) was 11%, while the disease control rate (DCR) was 77%. Although 35.2% of patients experienced grade 3 or higher immune-related adverse events, primarily hematological toxicities, no specific immune treatment-related hematological events were noted. Additionally, elevated D-dimer levels and hyponatremia emerged as prognostic factors associated with poorer outcomes, whereas hypertriglyceridemia correlated with enhanced survival. A nomogram developed for predicting PFS2 and OS demonstrated high discriminative capacity. **Conclusions:** These findings show that cross-line immunotherapy is safe and effective for patients with malignant tumors. It offers a viable option for patients who are progressing after initial treatments, which highlights the need for personalized strategies.

## 1. Introduction

In 2022, an estimated 20.0 million new cases and 9.7 million cancer deaths occurred worldwide [1]. Due to late-stage diagnoses, the majority of cancer deaths (56.1%) take place in Asia [1]. Notwithstanding advancements in conventional cancer treatments like surgery, chemotherapy, and targeted therapy, cancer persists as a public health threat and is the second leading cause of death worldwide [2]. Immunotherapy is an emerging therapeutic approach used to treat advanced or recurrent cancer and is considered a promising new approach [3]. Immune checkpoint inhibitors (ICIs) enhance the immune response against cancer cells by blocking immunosuppressive signals and are approved for various solid tumors. Despite the massive success of ICIs, durable antitumor responses and long-term remissions have been demonstrated only in a limited range of cancer types. For instance, in certain diseases such as melanoma, Hodgkin’s lymphoma, and MSI-high tumors, the response rate to ICIs in unselected patients is approximately 40% to 70%. However, in most other diseases, the response rate is limited to 10% to 25%, and the disease progression rate ranges from 10% to 70%, depending on the disease type [4]. Therefore, given the elevated risk of disease progression following ICIs, it is crucial to evaluate subsequent treatment options.

The mechanisms that cause drug resistance to ICIs remain unknown, which may be multifactorial. Although much focus has been on tumor factors such as PD-L1 expression, mutation burden, and antigen presentation deficiencies, the issue of immunotherapy resistance is more complex [5]. Hence, there is an immediate necessity for multimodal approaches targeting resistance mechanisms in immunotherapy. Various approaches are being explored to overcome resistance and enhance responses to ICIs. Regrettably, patients with disease progression post-ICI treatment have few treatment options available. Given the evolving immune response and the sustained benefits of ICIs, retreatment with the same or a different ICI (cross-line immunotherapy) appears to be an appropriate therapeutic strategy. This study aims to evaluate the clinical efficacy and safety of cross-line immunotherapy in patients with malignant tumors through a retrospective analysis and to identify practical clinical predictive markers.

## 2. Materials and Methods

### 2.1. Study Design and Patient Population

This retrospective study recruited patients with malignant tumors treated at the Cancer Center of Beijing Friendship Hospital from 1 January 2017 to 30 June 2023. The follow-up period ended upon the patient’s death, loss to follow-up, or 30 June 2024. We defined cross-line immunotherapy as a therapeutic approach in which patients who have experienced disease progression following initial or maintenance treatment with ICIs were retreated with the same or different ICIs, regardless of concurrent use with other therapeutic modalities, including chemotherapy or targeted therapy.

Patients included in the analysis were 18 years or older and had pathologically confirmed malignancies. They received at least two cycles of cross-line immunotherapy and had evaluable imaging data. Exclusion criteria were as follows: a documented family history of cancer; comorbidities such as autoimmune diseases or severe internal diseases including, but not limited to, cardiovascular conditions (e.g., atrioventricular block, atrial fibrillation, congestive heart failure), liver conditions (e.g., cirrhosis, hepatitis B), and kidney diseases requiring hemodialysis; lack of data on efficacy assessment, treatment discontinuation, or duration of the first immunotherapy; discontinuation of the initial immunotherapy due to toxic side effects followed by retreatment with the same regimen; inability to evaluate or lack of evaluation of cross-line immunotherapy; and loss to follow-up with less than three months of cross-line immunotherapy.

The primary endpoint of this study is progression-free survival on subsequent therapy (PFS2), defined as the interval from the commencement of cross-line immunotherapy to tumor progression. This study-specific definition of PFS2 differs from the conventional use of the term in oncology trials (which typically measures time from initial randomization to second disease progression). We employed this operational definition to assess the efficacy of the cross-line immunotherapy intervention directly. PFS1 was defined as the time from the initiation of first-line immunotherapy to the first objective disease progression. The other primary endpoint is overall survival (OS), the time from the start of treatment until death from any cause. Additionally, we calculated the objective response rate (ORR) and the disease control rate (DCR) according to RECIST 1.1 criteria. ORR refers to the percentage of cases achieving complete response (CR) or partial response (PR), whereas DCR includes cases achieving CR, PR, or stable disease (SD).

### 2.2. Data Collection

In this study, we recorded the fundamental characteristics and clinical data of all 105 patients who underwent cross-line immunotherapy, including age, sex, Eastern Cooperative Oncology Group (ECOG) performance status, tumor type, TNM staging, efficacy assessments of the initial and cross-line immunotherapy, treatment frequency, and details of combined therapies.

We also collected laboratory data from all patients within 7 days prior to the initiation of cross-line immunotherapy, including complete blood counts, hepatic and renal function tests, cardiac enzymes, lipid profile, coagulation profile, and tumor markers.

Medical records meticulously documented the patients’ fundamental characteristics and treatment details, including the timing of cross-line immunotherapy initiation, assessments of therapeutic efficacy, the time of disease progression or treatment failure, and immune-related adverse events.

Efficacy assessments were performed following RECIST 1.1 criteria, classifying responses as CR, PR, SD, or progressive disease (PD). The assessment of immune-related adverse events (irAEs) followed the National Comprehensive Cancer Network (NCCN) guidelines for managing toxicities related to ICIs. The severity of irAEs was graded using comprehensive medical records and objective laboratory data.

### 2.3. Statistical Analysis

Data were collected and managed using EpiData software (version 3.1, EpiData Association, Odense, Denmark). Statistical analyses and graphical representations were performed using R software, version 4.2.2. Measurement data conforming to the normal distribution were expressed as mean ± standard deviation, and non-normally distributed data were expressed as median (interquartile range). Enumeration data were expressed as a rate or percentage. For continuous variables, optimal prognostic cut-off values were determined using the ‘surv_cutpoint’ function from the ‘survminer’ R package. This function employs the maximally selected rank statistics method, which systematically evaluates all potential cut-points to identify the value that maximizes the separation between survival curves (i.e., the most significant log-rank statistic). For clinical interpretation, the statistically optimal cut-off was also compared to established laboratory reference ranges. We performed univariate and multivariate Cox regression analyses using the ‘rms’ package in R. Then, nomograms were constructed. We evaluated the model’s fit using time-dependent ROC curves, calculating the C-index, and employing calibration curves. Finally, we constructed a DCA curve for further assessment. *p* value < 0.05 (two-sided) indicated a statistically significant difference.

## 3. Results

### 3.1. Patient Characteristics

Ultimately, 105 patients were enrolled and met the inclusion and exclusion criteria. Most patients (two-thirds) were male, with a median age of 63 years (range 35–84). The median number of cross-line immunotherapy treatments received was five (range 2–34). Before cross-line immunotherapy, 51 patients had new distant metastases. At the time of treatment, 72 patients were on corticosteroids. Specific results are detailed in Table 1 and Table S1.

### 3.2. Efficacy

Of the 105 patients treated, 12 (11%) achieved PR, and 69 (66%) had SD; the remainder were classified as PD following the initial evaluation of cross-line immunotherapy with or without combination therapies. The ORR was 11%, and the DCR was 77% among the enrolled patients. The median follow-up duration was 9.9 months. The median cross-line immunotherapy PFS2 was 6.9 months (95% CI, 4.7–8.1), and the mOS was 12.9 months (95% CI, 11.7–NA). The PFS2 rates at 3, 6, and 12 months were 73.5% (95% CI, 0.654–0.826), 52.4% (95% CI, 0.434–0.634), and 27.0% (95% CI, 0.193–0.378), respectively. The OS rates at the same time points were 93.9% (95% CI, 0.893–0.988), 77.5% (95% CI, 0.695–0.865), and 56.3% (95% CI, 0.468–0.678), respectively.

To further investigate the impact of the specific “cross-line” strategy, we stratified patients according to whether the same ICI agent was used upon retreatment (Supplementary Table S2). Among the 105 patients, 52 (49.5%) received ICIs with change, while 53 (50.5%) were treated with ICIs without change. The mOS was 15.9 months (95% CI: 12.9–NA) in the ICIs with change group and 11.9 months (95% CI: 8.0–NA) in the ICIs without change group (*p* = 0.126) (Supplementary Figure S1). The mPFS2 was 7.0 months (95% CI: 5.9–9.8) and 4.9 months (95% CI: 3.9–9.1) in the change and without change groups, respectively (*p* = 0.676) (Supplementary Figure S2).

### 3.3. Optimal Cut-Off Values for Baseline D-Dimer, CEA, CRP, and LDH

We categorized continuous variables using cut-off values derived from statistical methods and clinically normal test values to facilitate analysis. The optimal cut-off values derived from statistical analysis for the baseline markers were as follows: D-dimer, 0.64 μg/mL; carcinoembryonic antigen (CEA), 11.4 ng/mL; C-reactive protein (CRP), 13.77 mg/L; and lactate dehydrogenase (LDH), 255 U/L.

### 3.4. Prognostic Factors for PFS2 and OS

**Figure 1:**
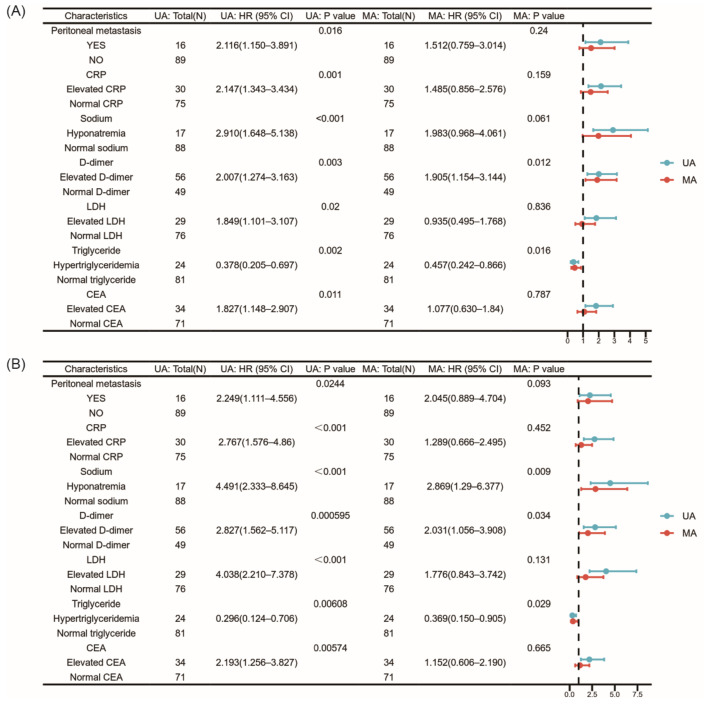
Forest plot of univariate and multivariate Cox regression analyses of risk factors related to cross-line immunotherapy. (**A**) The forest plot for PFS2. (**B**) The forest plot for OS.UA: univariate analyses, MA: multivariate analyses, and CEA: carcinoembryonic antigen.

Initially, we performed a univariate analysis of all clinical factors, setting a *p*-value threshold of 0.05 for selection. Subsequent optimization with the stepwise method yielded a final multivariate COX regression model that included seven variables: peritoneal metastasis, hyponatremia, elevated CRP, elevated D-dimer, elevated triglycerides, elevated LDH, and elevated CEA. Recognizing the diversity of tumor types and the potential for statistical bias due to small sample sizes in some tumor types, we stratified the patient cohort into lung cancer (39/105), digestive tumors (47/105), and other malignancies (19/105) based on the sample size of each tumor type. In a stratified, multifactorial COX regression analysis, our study indicated that elevated D-dimer levels were associated with a poor prognosis for both PFS2 and OS. In contrast, high triglyceride levels, also known as hypertriglyceridemia, were associated with a better prognosis for both PFS2 and OS. Moreover, hyponatremia was detrimental to OS prognosis but did not significantly affect PFS2. The other clinical variables did not statistically correlate with PFS2 or OS. Detailed results are presented in Figure 1.

### 3.5. Kaplan–Meier Survival Curve Analysis

**Figure 2:**
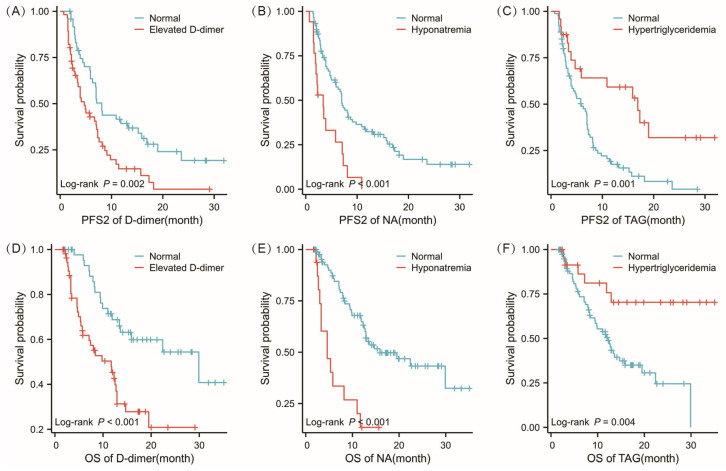
The Kaplan–Meier plots of PFS2 and OS for patients with cross-line immunotherapy stratified by various risk factors. (**A**–**C**) Survival curve analysis showed the PFS rates of different groups stratified by D-dimer, hyponatremia, and hypertriglyceridemia. (**D**–**F**) Kaplan–Meier curves of OS in D-dimer, hyponatremia, and hypertriglyceridemia groups.

Multivariate Cox regression analysis revealed that elevated D-dimer levels, hyponatremia, and hypertriglyceridemia were independent prognostic factors in patients undergoing cross-line immunotherapy. We also generated survival curves for PFS2 and OS for these factors (Figure 2) and performed log-rank tests to evaluate the significance of these differences. The results indicated that the median PFS2 was significantly shorter in the elevated D-dimer group than in the normal group, at 4.7 months (95% CI: 3.4 to 7.3) versus 8.1 months (95% CI: 6.3 to 15.9). The median OS was also shorter in the elevated D-dimer group, at 11.7 months (95% CI: 5.8 to 12.9) compared to the normal group’s 29.9 months (95% CI: 13.6 to not reached). In the hyponatremia group, the mPFS2 was 3.4 months (95% CI: 2.0 to 7.3), whereas the normal group had an mPFS2 of 7.0 months (95% CI: 5.9 to 9.8). There was also a significant difference in the median OS between the hyponatremia and normal groups, with 4.6 months (95% CI: 3.3 to 11.7) versus 15.9 months (95% CI: 12.7 to not reached). The mPFS2 for the hypertriglyceridemia group was 16.9 months (95% CI: 5.9 to beyond estimation), longer than the 5.9 months observed in the normal group (95% CI: 3.9 to 7.1), with a statistical difference (*p* = 0.001). However, the median OS was not reached, while it was 12.3 months in the normal group (95% CI: 9.4 to 15.9), with a significant difference between the two groups (*p* = 0.004).

### 3.6. Nomogram for Survival Prediction

**Figure 3:**
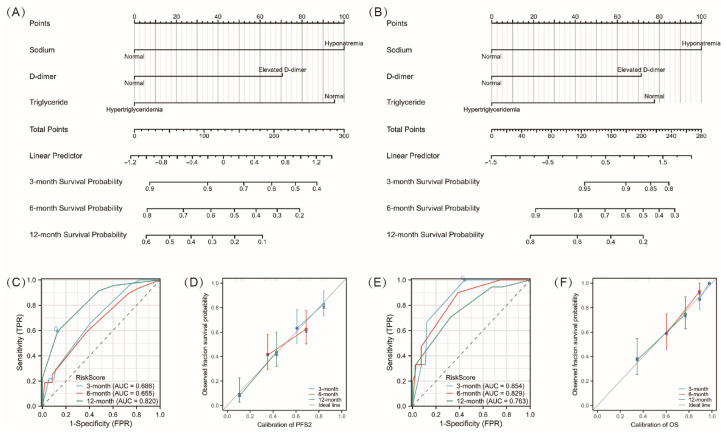
Construction and validation of a nomogram for PFS2 and OS. (**A**) Nomogram to predict the 3-, 6-, and 12-month PFS2. (**B**) Nomogram to predict the 3-, 6-, and 12-month OS. (**C**,**D**) Time-dependent ROC curves and Calibration curves for the nomogram model of PFS2. (**E**,**F**) Time-dependent ROC curves and Calibration curves for the nomogram model of OS.

A nomogram model was developed to predict PFS2 and OS rates at 3, 6, and 12 months following cross-line immunotherapy (Figure 3A,B). These nomograms utilized variables derived from multivariable Cox proportional hazards models: D-dimer, triglycerides, and hyponatremia for both PFS2 and OS models. Points were assigned to each variable based on the hazard ratios (HRs) derived from the Cox model.

**Figure 4:**
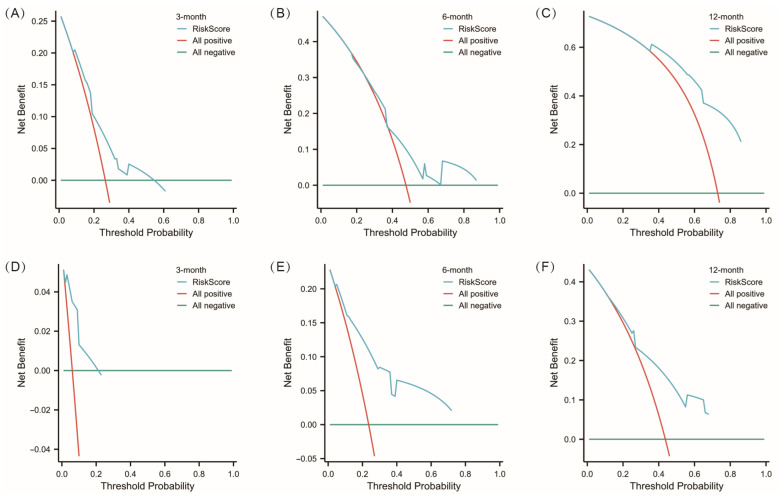
Decision curve analysis for the nomogram model. (**A**–**C**) Clinical impact curve to evaluate the clinical role of the Nomogram model for PFS2 in 3, 6, and 12 months. (**D**–**F**) Clinical impact curve to evaluate the clinical role of the Nomogram model for OS in 3, 6, and 12 months.

Various methods were used to evaluate nomogram discriminatory performance, such as time-dependent ROC curves, C-index values, calibration curves, and decision curve analysis (DCA). The nomogram’s time-dependent ROC curves for predicting PFS2 and OS at 3, 6, and 12 months were shown in Figure 3C,E, with AUC values for PFS2 at 3, 6, and 12 months being 0.686, 0.655, and 0.820, respectively; and for OS at 3, 6, and 12 months being 0.854, 0.829, and 0.763, respectively. Additionally, the C-index for our nomogram models predicting PFS2 at 3, 6, and 12 months was 0.683, 0.644, and 0.658, respectively. Correspondingly, the C-indexes for the OS nomogram models at the same time points were 0.845, 0.804, and 0.724, respectively, indicating high discriminative power for the models. We used calibration curves to visually illustrate the relationship between actual and predicted probabilities. Figure 3D,F show that the calibration curves, without apparent deviations from the reference line, illustrate satisfactory agreement between model predictions and actual observations for PFS2 at 3, 6, and 12 months. While the nomograms showed good agreement between predicted and observed OS at 6 and 12 months on the calibration curves, a minor discrepancy was noted in the prediction of OS at 3 months. Figure 4 demonstrates the decision curves for the same model at various time points. The decision curve indicated that when the threshold probability ranged from 0 to 1.0, our model, when predicting 6-month PFS2 and OS, provided greater net benefit than the “treat-all” or “treat-none” schemes. However, at other time points, the predictive model’s net benefit was lower than that of the “no treatment” strategy for certain threshold probabilities.

### 3.7. Safety Profiles

Among 105 patients, the overall incidence of treatment-related toxicities was 98.1%, and only two patients had no toxicities at any grade. The incidence of grade 3 or higher immune-related toxicities was about 41.0%, and it decreased to 35.2% after excluding cases of severe fasting hyperglycemia. The most common treatment-related adverse reactions were hematological toxicities (with an incidence of 77.1%), including leukopenia, neutropenia, anemia, and thrombocytopenia. Of these, 26.7% were grade 3 or higher. However, specific immune treatment-related hematological toxicities, such as aplastic anemia and acquired hemophilia, did not occur. One case was considered to have grade 4 cardiotoxicity, and the other case had grade 3 cardiotoxicity. Only one case of immune-related pneumonia was observed, and it was grade 1 in severity. No cases of immune-related neurotoxicity, ocular toxicity, musculoskeletal toxicity, or pancreatic toxicity were reported. For detailed results, see Table 2.

## 4. Discussion

Immunotherapy resistance remains a major clinical challenge. In this context, two main strategies for re-initiating immunotherapy after disease progression have been described: retreatment (re-administering the same agent after a therapy-free interval) and rechallenge (re-initiating immunotherapy after intervening with other therapies) [6,7]. Current evidence, however, is largely confined to these paradigms and predominantly derived from studies in melanoma and non-small cell lung cancer [8,9,10,11,12]. In contrast, there is a paucity of data focusing on a distinct, clinically pressing scenario, the strategy of “cross-line immunotherapy,” defined as continuing or switching ICIs immediately upon progression during initial immunotherapy. This strategy remains understudied, especially across a broader spectrum of solid tumors.

The rationale for switching to a different ICI after initial immunotherapy progression is based on clinical considerations and biological plausibility. In practice, switching may be prompted by the management of specific immune-related adverse events associated with the initial agent, or by drug availability and clinician/patient preferences in real-world care [13,14]. Mechanistically, even among ICIs targeting the PD-1/PD-L1 axis, differences in antibody structure, epitope binding, affinity, and Fc-mediated functions may lead to distinct activity and toxicity profiles [15,16], offering a potential basis for non-cross-resistance. The 77% disease control rate observed in our cohort with a switch strategy supports this concept. Nevertheless, the optimal treatment sequence and underlying biomarkers of response remain unclear and require validation in prospective, biomarker-driven trials.

A key consideration in interpreting our findings is the 91.4% of patients who received cross-line immunotherapy in combination with other therapies, primarily chemotherapy. It is well-established that combining immunotherapy with chemotherapy or antiangiogenic agents can have synergistic effects [17,18,19,20]. Therefore, the observed progression-free survival and overall survival benefits may reflect a synergistic effect between the ICI and the new chemotherapy backbone, rather than the effect of ICI continuation or switching alone. Similarly, the identified prognostic factors might be associated with a more aggressive disease biology that influences response to combined modality therapy in general, making it challenging to isolate predictors specific to ICI rechallenge. Future prospective studies stratifying patients by the type of concomitant therapy or incorporating randomized designs are warranted to disentangle the independent contribution of the ICI component.

Historical data show that when immunotherapy is initially used, mPFS varies significantly across tumor types, ranging from 1.9 to 26.1 months. The mOS ranges from 8.2 months to over 36 months, and the ORR ranges from 10.9% to 79% [21]. Scheiner et al. prospectively investigated the application of immunotherapy rechallenge in liver cancer, with an mOS of 12.0 months, achieving an ORR of 26% and a DCR of 55% [22]. A meta-analysis of immunotherapy retreatment for non-small cell lung cancer and melanoma demonstrated a DCR ranging from 22% to 83%, with an mOS of 12 to 20.6 months [12]. A different meta-analysis on lung cancer found that the overall ORR for patients undergoing immunotherapy retreatment was 20%, while the DCR was 54% [8]. Compared with these data, our study validated the efficacy of cross-line immunotherapy (mPFS was 6.9 months, mOS was 12.9 months) and confirmed its potential as an effective treatment option. Moreover, our subgroup analysis revealed that clinical benefit was observed regardless of whether a different or the same ICI agent was used during cross-line treatment (median PFS2: 7.0 vs. 4.9 months; median OS: 15.9 vs. 11.9 months), although the differences were not statistically significant. This suggests that the potential efficacy of the cross-line immunotherapy strategy is not affected by whether the ICIs are changed.

In addition to efficacy, safety is a significant concern in cross-line immunotherapy. ICIs enhance antitumor responses by activating the patient’s immune system, but they may also trigger irAEs [23,24]. These adverse events can affect multiple systems, including the skin, gastrointestinal tract, endocrine system, and lungs, and may be fatal in severe cases [25,26]. Yang et al. conducted a meta-analysis to evaluate the occurrence of irAEs after retreatment with different ICIs. For patients who received CTLA-4 inhibitors during retreatment, the incidence of grade 3 irAEs ranged from 5.9% to 25%. In patients retreated with PD-1 inhibitors, the incidence of grade 3 irAEs was less than 10%. In patients treated with PD-L1 inhibitors, the incidence of grade 3 irAEs ranged from 0% to 15%. In contrast, patients receiving combination therapy with ICIs and other drugs had a higher incidence of grade 3 irAEs, ranging from 0% to 64% [12]. Guo et al. [10] reported that immunotherapy rechallenge can increase the risk of irAEs. In their findings, 60% of patients experienced a recurrence of existing irAEs or developed new ones. Furthermore, 50% of these patients progressed to grade 2 or higher irAEs. In our study, the incidence of adverse events grade 3 or higher was 35.2% after excluding hyperglycemia. The most common adverse events were hematological toxicities, accounting for 77.1%, including leukopenia, neutropenia, anemia, and thrombocytopenia, possibly linked to the widespread use of combination therapies, especially cytotoxic chemotherapy. However, no specific hematologic toxicities, such as aplastic anemia, were associated with immunotherapy in purists. The study recorded one case of grade 4 severe cardiotoxicity and another case of grade 3 cardiotoxicity. Both patients had a history of coronary heart disease and diabetes. Only one case of grade 1 immune-related pneumonia was reported. No cases of immunotherapy-related neurotoxicity, ocular toxicity, musculoskeletal toxicity, or pancreatic toxicity arose from the use of cross-line immunotherapy. The study reported a low incidence of immune-related toxicities, with most patients tolerating treatment well. This tolerance may be linked to the absence of severe immune-related adverse events during the initial immunotherapy in the selected patients.

Despite ongoing clinical efforts to identify biomarkers that predict immunotherapy efficacy, such as tumor mutational burden and PD-L1 expression, these markers have significant limitations in terms of accuracy and practical application [27,28,29]. After disease progression in patients with advanced cancer, clinical problems such as poor adherence and difficulty in obtaining tissue samples can make it more challenging to obtain biomarkers such as tumor mutational load (TMB) and PD-L1 expression. Therefore, developing practical, non-invasive predictors to identify patients suitable for cross-line immunotherapy is particularly important. Our study investigated clinical predictors of cross-line immunotherapy and found that D-dimer, hyponatremia, and hypertriglyceridemia are associated with prognosis, leading to the development of a predictive model. Previous studies [30,31,32] have confirmed that hyponatremia is a prognostic marker for poor treatment outcomes in malignant tumors during chemotherapy or immunotherapy, and our study further confirms the potentially persistent adverse effects of hyponatremia throughout the treatment process for these cancers. D-dimer is also considered a marker of poor prognosis in malignant tumors and may be associated with deep vein thrombosis and pulmonary embolism [33,34,35]. While our study supports the correlation between D-dimer and poor prognosis, it does not conclusively determine the presence of thrombosis in patients. Therefore, future studies should assess thrombosis in patients with high D-dimer levels and may explore preventive treatments. We acknowledge that D-dimer and hyponatremia are well-established, non-specific markers of systemic inflammation and poor general condition in advanced cancer [36,37]. Their strong association with outcomes in our cohort likely reflects, in part, the selection of patients with more aggressive disease or poorer performance status for cross-line therapy.

Hypertriglyceridemia, acting as a protective factor, provides results that are contrary to clinical expectations. Our study found that total cholesterol, low-density lipoprotein (LDL), and high-density lipoprotein (HDL) cholesterol do not affect treatment outcomes. This may be because diet significantly influences triglyceride levels [38]. Nevertheless, evidence indicates the essential role of lipid metabolism in immune regulation, with triglyceride levels suggesting a more direct involvement. For example, Andersen’s research indicated that, in autoimmune diseases, elevated triglyceride levels have a higher predictive value for antinuclear antibody status than total cholesterol, low-density lipoprotein cholesterol, and high-density lipoprotein cholesterol [39]. Ali et al.’s study demonstrated that macrophages can release pro-inflammatory cytokines under high triglyceride conditions, thereby confirming that high triglyceride levels affect immune response regulation [40]. A study found that specific triglyceride species were significantly lower in patients who developed irAEs, suggesting that these lipids may serve as early biomarkers of irAE risk [41]. This points to a potential link between lipid metabolism and immune-related toxicity, underscoring the need for metabolomic profiling in cancer immunotherapy. Therefore, we hypothesize that elevated triglyceride levels may not only reflect a better nutritional status, which can influence systemic metabolism, but also a more direct role in immune modulation. Hypertriglyceridemia may be a surrogate marker for a specific immunometabolic phenotype that influences responses to immunotherapy, warranting further investigation.

In addition, a single biomarker is unlikely to predict patient outcomes treated with ICIs [42,43]. In this retrospective study, we built a Cox regression model and a nomogram to identify patients most likely to benefit from cross-line immunotherapy. Our nomogram model integrates various clinical indicators identified by COX regression analysis, effectively distinguishing patients with different risk levels. Time-dependent ROC curves, C-indices, and calibration plots assess the model’s accuracy. ROC curve analysis for PFS2 and OS at 3, 6, and 12 months showed that the model’s AUC values exceeded 0.6, indicating good discriminatory power.

Furthermore, the C-index for PFS2 exceeded 0.65, and the C-index for OS ranged from 0.724 to 0.848, further validating the model’s reliability and practicality. The calibration plot results showed no significant deviation between the model’s predicted values and actual observations, indicating satisfactory agreement between the model’s predictions and actual outcomes. We also generated a DCA to assess the net benefit of prediction-assisted decisions. DCA shows that our nomogram has a higher overall net benefit for predicting survival in patients receiving cross-line immunotherapy across all threshold probabilities.

Cross-line immunotherapy shows promise as a salvage strategy for patients with malignant tumors progressing after initial immunotherapy, particularly those with favorable prognostic markers identified in this study: hypertriglyceridemia, absence of hyponatremia, and normal D-dimer levels. Its application may be prioritized for patients who previously demonstrated clinical benefit from initial immunotherapy and tolerate treatment without severe irAEs. The high disease control rate (77%) and manageable safety profile support its use in advanced solid tumors, including non-small cell lung cancer and digestive malignancies. Conversely, contraindications include pre-existing severe irAEs, uncontrolled autoimmune diseases, and laboratory markers indicating poor prognosis (elevated D-dimer, hyponatremia). Further prospective studies are needed to validate these indicators and refine patient selection criteria.

The limitations of this study warrant careful consideration. The single-center retrospective design inherently introduces biases related to patient selection and confounding variables that may affect treatment outcomes. The most significant limitation is the substantial heterogeneity of our cohort, which included over 15 tumor types with diverse biologies and immunotherapeutic sensitivities. Furthermore, treatment regimens for cross-line therapy were not standardized. They involved various immune checkpoint inhibitors used in different lines and, in some cases, combined with other agents. Although stratified, the small subgroup sample sizes limit the interpretability of the aggregated result (e.g., mPFS2 of 6.9 months). It remains uncertain whether this reflects a broad effect or is driven by responsive subsets, such as NSCLC. Therefore, our findings should be considered hypothesis-generating, underscoring the necessity for future prospective, tumor-specific studies with larger cohorts to definitively evaluate the efficacy of this cross-line immunotherapy strategy.

## 5. Conclusions

In conclusion, our research provides strong evidence for the effectiveness and safety of cross-line immunotherapy in patients with malignant tumors who have experienced disease progression. We identified key prognostic factors, including elevated D-dimer levels, hyponatremia, and hypertriglyceridemia. These factors suggest the possibility of tailoring treatment strategies to individual patients. These findings not only enhance the current understanding of treatment options but also carry significant clinical implications, helping to shape future therapeutic strategies and ultimately improving patient outcomes in oncology.

## Figures and Tables

**Table 1:** Patient baseline clinical characteristics.

Characteristics	*N* (%)	Characteristics	*N* (%)
Age at Start of Cross-line Immunotherapy		Cancer Diagnosis (Continued)	
Median	63 (35–84)	EC	3 (2.9)
≥60 years	70 (66.7)	CC	2 (1.9)
<60 years	35 (33.3)	OC	1 (1)
Sex		Melanoma	2 (1.9)
Male	74 (70.5)	HNSCC	3 (2.9)
Female	31 (29.5)	Other Cancers	4 (3.8)
ECOG Performance Status		Cancer Stage	
0	27 (25.7)	II	1 (1)
1	74 (70.5)	III	13 (12.4)
2	4 (3.8)	IV	88 (83.8)
Cancer Diagnosis		Unknown	3 (2.9)
NSCLC	30 (28.6)	New Distant Metastasis	
SCLC	9 (8.6)	Yes	51 (48.6)
EC	13 (12.4)	No	54 (51.4)
GC	17 (16.2)	Type of Cross-line Immunotherapy	
CRC	5 (4.8)	PD-1	98 (93.3)
HCC	6 (5.7)	PD-L1	7 (6.7)
PC	1 (1)	Concurrent Treatment	
BTC	5 (4.8)	Yes	96 (91.4)
RCC	1 (1)	No	9 (8.6)
UC	2 (1.9)	Cross-line Treatment Episodes	
HL	1 (1)	Median (Range)	5 (2–34)

NSCLC = Non-Small Cell Lung Cancer, SCLC = Small Cell Lung Cancer, EC = Esophageal Cancer, GC = Gastric Cancer, CRC = Colorectal Cancer, HCC = Hepatocellular Carcinoma, PC = Pancreatic Cancer, BTC = Biliary Tract Cancer, RCC = Renal Cell Carcinoma, UC = Urothelial Carcinoma, HL = Hodgkin’s Lymphoma, EC = Endometrial Cancer, CC = Cervical Cancer, OC = Ovarian Cancer, MEL = Melanoma, HNSCC = Head and Neck Squamous Cell Carcinoma.

**Table 2:** Immunotherapy-related adverse events.

	Overall Populations (*N* = 105)	
	All Grades	Grade ≥ 3
Hyperglycemia	67 (63.8)	13 (12.4)
White blood cell count decreased	64 (61.0)	12 (11.4)
Anemia	62 (59.0)	16 (15.2)
Neutrophil count decreased	49 (46.7)	14 (13.3)
Bilirubin increased	37 (36.3)	8 (7.6)
Platelet count decreased	37 (36.3)	5 (4.8)
Aspartate aminotransferase increased	31 (29.5)	5 (4.8)
Alanine aminotransferase increased	29 (27.6)	3 (2.9)
Creatinine increased	12 (11.4)	0
Hypothyroidism	7 (6.7)	0
Pruritic	5 (4.8)	0
Cardiotoxicity	4 (3.8)	4 (3.8)
Diarrhea/colitis	4 (3.8)	0
Rash	3 (2.9)	1 (1.0)
Capillary vasodilation syndrome	3 (2.9)	0
Hyperthyroidism	2 (1.9)	0
Reaction to infusion	1 (1.0)	1 (1.0)
Pneumonitis	1 (1.0)	0

## Data Availability

The data presented in this study are available on request from the corresponding author due to the detailed and sensitive personal information of the participants.

## Supplementary Materials

The following supporting information can be downloaded at https://www.mdpi.com/article/10.3390/jcm15072751/s1, Figure S1: The Kaplan-Meier plot of OS stratified by ICIs change status; Figure S2: The Kaplan-Meier plot of PFS2 stratified by ICIs change status; Table S1: Comprehensive patient baseline clinical characteristics; Table S2: Distribution of cross-line immune checkpoint inhibitor switching patterns.
