# Supplementary material for: Navigating Immunotherapy Resistance: The Role of Cross-Line Strategies in Cancer Treatment
Source: J Clin Med. 2026 Apr 5;15(7):2751. doi: 10.3390/jcm15072751 (PMC13073642; doi:10.3390/jcm15072751)

Supplementary Figure S1 The Kaplan-Meier plot of OS stratified by ICIs change status

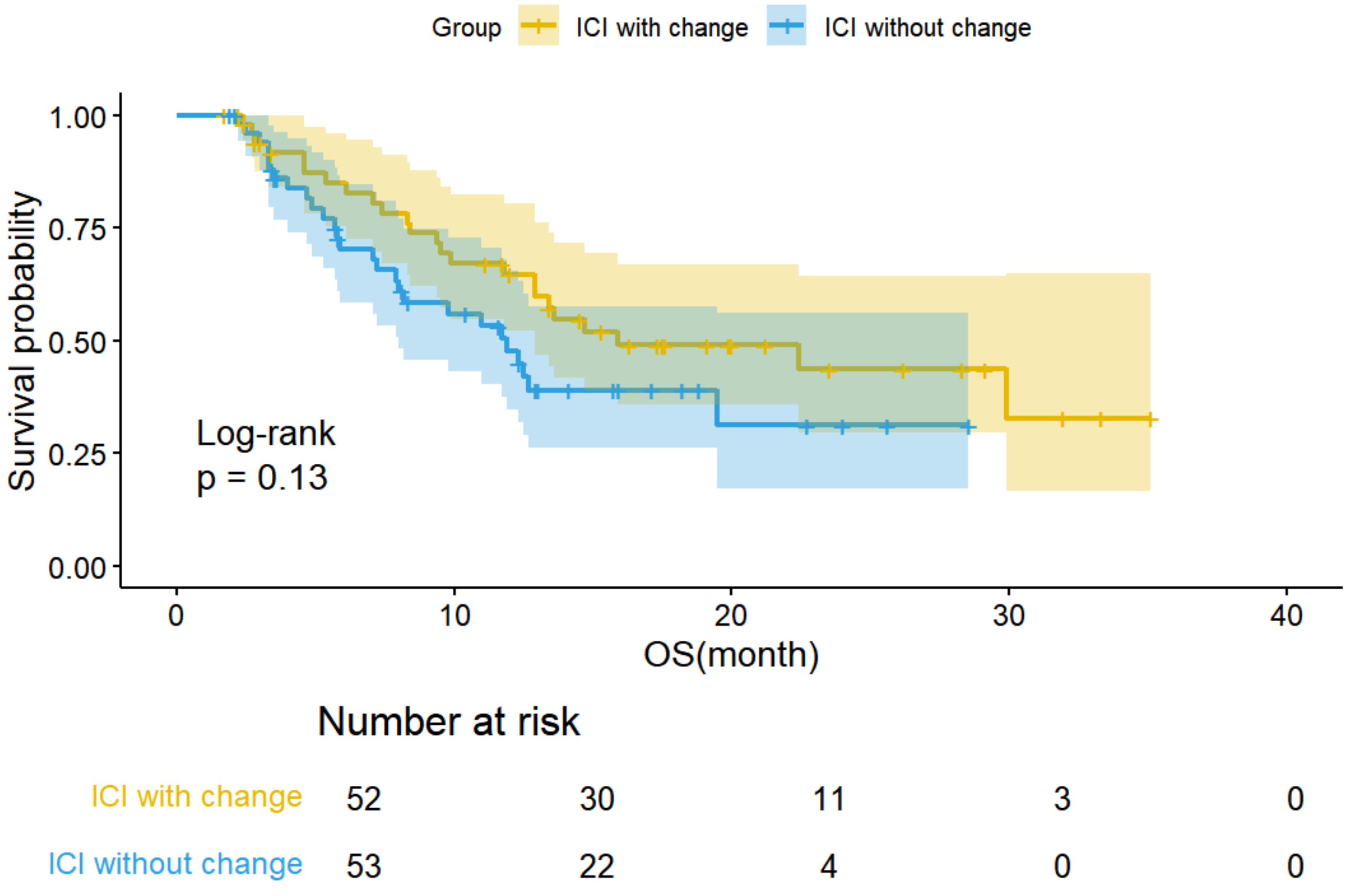

Supplementary Figure S2 The Kaplan-Meier plot of PFS2 stratified by ICIs change status

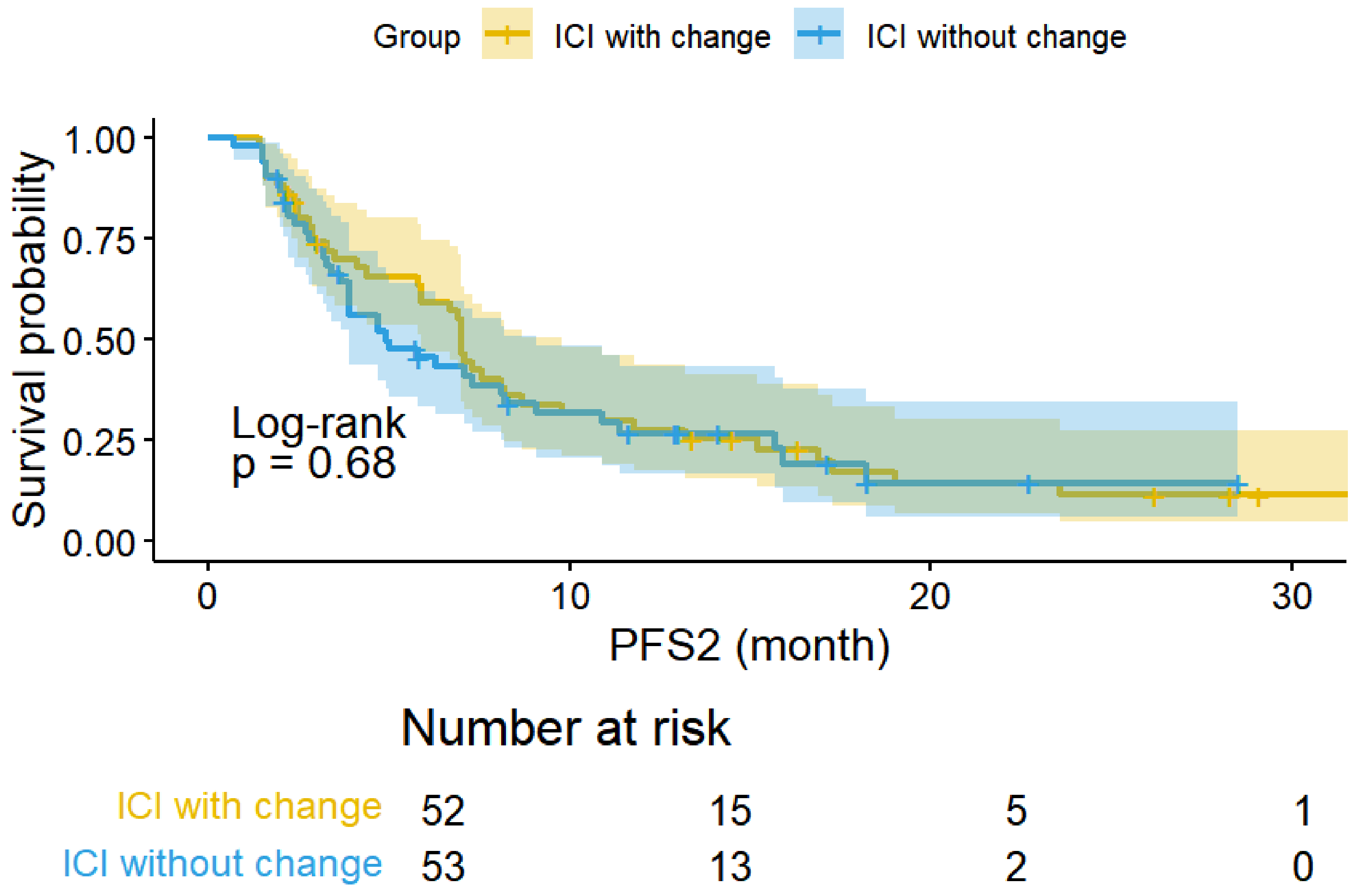

Supplement: Supplementary file 1 [file jcm-15-02751-s001.zip › Supplementary Figures.pdf]
